# Phosphorus-based Flame Retardancy Mechanisms—Old Hat or a Starting Point for Future Development?

**DOI:** 10.3390/ma3104710

**Published:** 2010-09-30

**Authors:** Bernhard Schartel

**Affiliations:** BAM Federal Institute for Materials Research and Testing, Unter den Eichen 87, 12205 Berlin, Germany; E-Mail: bernhard.schartel@bam.de; Tel.: +49 30 8104 1021; Fax: +49 30 8104 1617

**Keywords:** fire retardancy, red phosphorus, phosphate, phosphonate, phosphinate, phosphine oxide, flame inhibition, charring, intumescence

## Abstract

Different kinds of additive and reactive flame retardants containing phosphorus are increasingly successful as halogen-free alternatives for various polymeric materials and applications. Phosphorus can act in the condensed phase by enhancing charring, yielding intumescence, or through inorganic glass formation; and in the gas phase through flame inhibition. Occurrence and efficiency depend, not only on the flame retardant itself, but also on its interaction with pyrolysing polymeric material and additives. Flame retardancy is sensitive to modification of the flame retardant, the use of synergists/adjuvants, and changes to the polymeric material. A detailed understanding facilitates the launch of tailored and targeted development.

## 1. Introduction

Different kinds of additive and reactive approaches to compounds containing phosphorus are finding success, so that increasingly they are proposed as halogen-free flame retardants for various polymeric materials and applications with different mechanisms exploited and efficiencies achieved. In these, phosphorus can act in the condensed phase by enhancing char [1,2,3,4], by yielding intumescence [5,6,7,8], or through inorganic glass formation [9,10]; and in the gas phase through flame inhibition [1,11,12,13]. In fact, for many systems a significant action has been determined in both the gas and condensed phases [9,14,15,16,17,18]. The occurrence and the efficiency seems to depend not only on the chemical structure of the phosphorous flame retardant compound itself, but also on the interaction during pyrolysis with its chemical environment, including the polymeric material [9,18,19,20,21] and other additives. Indeed, it is assumed that the mechanism and efficiency of phosphorous flame retardants are influenced and can be optimized by modifying the flame retardant using synergists and adjuvants and by changing the polymeric material [1,19,22,23]. A detailed understanding to facilitate the launch of tailored or targeted development approaches is lacking.

This review indicates how to fill this gap using results and data of previously published own studies. First, some principles of the mechanisms are addressed in detail, followed by several examples based on systematically varied systems. These examples are classified into three different categories: Different flame retardants containing phosphorus are used in the same polymer; then the same phosphorus-containing flame retardant is used in different polymers; and finally phosphorus-containing flame retardants are used in combination with other flame retardants and synergists. By means of these systematic comparisons, the extensive and complex matrix of structure-property relationships is examined. Comprehensive thermal analysis is proposed to gain insight into the characteristics of pyrolysis, flammability tests and cone calorimeter, to characterize flame retardancy. The focus is on the flame-retardancy mechanisms, and in particular, on how they are controlled by the decomposition pathways and interactions occurring during pyrolysis. The flame retardancy mechanisms and effects achieved are assessed in order to work out promising or tailored flame retardancy approaches.

## 2. Results and Discussion

### 2.1. Flame Retardancy Mechanisms

Since the understanding of flame retardancy mechanisms of flame retardants containing phosphorus tends to be oversimplified, in this subsection some details are underlined. Some general points about fire retardancy mechanisms will be clarified in advance, with the following subsection concentrating on more specific results and conclusions with respect to the tasks and materials investigated.

In a very similar way to the flame retardants containing halogen; those containing phosphorus can act in the gas phase [11,24]. In this case hydrogen and hydroxy radicals are replaced by less effective radicals or are rendered harmless by radical recombination in the gas phase. Some key reactions, of the hundreds possible, are proposed to be the most important (Equation 1). Branching and chain reactions of the oxidation of hydrocarbons in the gas phase are slowed down or interrupted, which is called flame inhibition, and reduces the production of heat. The efficiency of P in the gas phase is reported to be similar or even superior to hydrogen halides like HBr [25]. Although detailed investigations of the flame zone, such as identifying the intermediate products and monitoring concentrations of the different products, are rather rare [11,26,27,28], the main principle seems to be understood. It is believed that the PO-radical plays the major role. Furthermore, the resulting flame retardancy effects are obvious, including a clearly decreased heat release due to a reduced heat release rate (HRR)/mass loss rate value during flammability and fire tests.

(1) PO•+H•→HPO

$$PO•+OH•\to HPO_{2}$$

$$HPO+H•\to H_{2}+PO•$$

$$OH•+H_{2}+PO•r\to H_{2}O+HPO$$

$$HPO_{2}•+H•\to H_{2}O+PO$$

$$HPO_{2}•+H•\to H_{2}+PO_{2}$$

$$HPO_{2}•+OH•\to H_{2}O+PO_{2}$$

However, with respect to the gas phase flame retardancy action of phosphorus, in this review two general aspects are pointed out that are often overlooked but are worth addressing. First of all, the major prerequisite of an effective gas phase mechanism is that suitable phosphorus-containing compounds are volatile during polymer pyrolysis. This is not as trivial as it sounds, since in the condensed phase many phosphorus-containing flame retardants show decomposition and/or chemical reactions with each other or other compounds, and their decomposition products may react into volatile or solid products depending on the chemical surroundings in the pyrolysis zone. Hence, even for systems in which phosphorus works in the gas phase, understanding pyrolysis in the condensed phase is the key to understanding the flame retardancy mechanism. Secondly, flame inhibition leads to a less complete combustion in the flame zone, as this effect is specific to a distinct fire scenario. Generally, in a distinct fire test, flame inhibition decreases the combustion efficiency χ, but not the specific effective heat of combustion of the volatiles (h_c_(volatiles)). The latter is reduced only through an often negligible dilution effect as carbon is replaced by P. However, the product χ•h_c_ is reduced and hence also the heat release. Or in other words, flame inhibition is more or less equal to a slow down of the oxidation within the flame zone. Assuming a similar or reduced finite size of the flame, and thus an equal or reduced finite time period for the oxidation of the released unchanged pyrolysis products, an incomplete oxidation occurs. The heat release of infinite flame zones and infinite time periods would not be influenced by flame inhibition. 

The effect on χ of adding aluminium tris-(diethylphosphinate) (AlPO2) to poly(butylene terephthalate) (PBT) and PBT reinforced with glass fibers (PBT-GF) in the cone calorimeter test is shown in Table 1 as an example. 

**Table 1:** Reduction of the combustion efficiency χ in the cone calorimeter (irradiation 50 kWm^–2^) through flame inhibition.

Material	P/(PBT+AlPO2)	THE/TML	h_c_(volatiles)	χ
	in wt.-%	kJg^–1^	kJg^–1^	(kJg^–1^)/(kJg^–1^)
		+/−0.7	+/−0.4	+/−0.07
PBT	0	20.7	20.0	1.00
PBT/30 wt.-% GF	0	19.4	20.0	0.97
PBT/6.3 wt.-% AlPO2	1.50	16.6	19.8	0.84
PBT/6.5 wt.-% AlPO2	1.55	16.0	20.3	0.79
PBT/30 wt.-% GF/ 4.4 wt.-% AlPO2	1.50	14.1	20.0	0.71
PBT/30 wt.-% GF/ 6.3 wt.-% AlP O2	2.14	12.8	19.9	0.64
PBT/20 wt.-% AlPO2	4.76	13.2	20.5	0.65
PBT/30 wt.-% GF/ 14 wt.-% AlPO2	4.76	13.5	20.3	0.67
PBT/30 wt.-% GF/ 20 wt.-% AlPO2	6.80	14.8	20.4	0.73

It has previously been reported that AlPO2 acts in PBT and PBT-GF mainly through flame inhibition [29,30,31]. And indeed, dividing the cone calorimeter results for the total heat evolved/total mass loss (THE/TML) through the h_c_(volatiles) determined through the pyrolysis combustion flow calorimeter (PCFC) deliver χ around 1 for PBT and PBT-GF and clearly reduced χ for the flame retarded materials. The result for PBT and PBT-GF corresponds to the well ventilated fire scenario of the cone calorimeter [32]. The flame inhibition by released compounds containing phosphorus are characterized by the reduced χ, whereas h_c_(volatiles) is hardly influenced.

Reducing the combustion efficiency is usually accompanied by an increase in CO and smoke production, both typical products of incomplete combustion. The conversion of CO to CO_2_ is typically the main exothermic reaction [33] and contributes crucially to heat production in the flame. Thus efficient flame inhibition is intrinsically accompanied by an increase in CO yield.

The phosphorus remaining in the condensed phase also reduces fire risks, mainly when the charring of the polymer is enhanced or inorganic glasses are formed. The general mechanism is understood in part [1,2,3,33,34,35]: Dehydration of the polymeric structure, which induces cyclization, cross-linking, aromatization/graphitization; cross-linking for which phosphorus compounds or their decomposition products act as cross-linkers; and the formation of inorganic glasses such as polyphosphates, but probably also phosphoric acid. However, with respect to the condensed phase flame retardancy action of phosphorus, this work maps out or elaborates general aspects worthy of particular attention. 

First of all, not only the decomposition of flame retardants containing phosphorus, but also their interactions and reactions with the chemical surroundings in the pyrolysis zone, control the condensed phase mechanisms. This is not as trivial as it sounds. Indeed, small changes in the system can result in major changes in the mechanisms, which may be overseen in the discussion. For instance, 12.5 wt.-% bisphenol A bis(diphenyl phosphate) (BDP) was reported to work differently in bisphenol A poly(carbonate) (PC_PTFE_) than in 71 wt.-% PC/15 wt.-% acrylonitrile-butadiene-styrene blends (PC_PTFE_/ABS/BDP) [36]. The materials contain ~0.4 wt.-% poly(tetrafluoroethylene) (PTFE) as an anti-dripping agent, which is indicated by the subscript. In PC_PTFE_, the exclusive release of products containing phosphorus results in flame inhibition. In PC_PTFE_/ABS, a release of phosphorous and thus flame inhibition occurs, and P remains in the condensed phase, enhancing PC charring (Table 2). 

**Table 2:** Charring of BDP in PC_PTFE_/BDP and PC_PTFE_/ABS/BDP. The residues are determined by thermal analysis (heating rate 10 K min^–1^) under nitrogen right after the decomposition step (~820 K).

	Residue	Expected residue	Additional carbonaceous charring
PC (/PC in PC/ABS)	31.5 (/27.2)%		
BDP	4.2%		
PTFE	0.4%		
ABS	3.2%		
PC_PTFE_/BDP	27.9%	28.0%	no
PC_PTFE_/ABS	22.7%	26.7%	no
PC_PTFE_/ABS/BDP	25.2%	19.4 (–23.8*)%	yes

* a residue range is indicated to account for the different char yields of PC in PC and PC/ABS.

Interaction between BDP and the early-stage decomposition products, such as Fries rearranged carbonate structures of PC, was proposed in the condensed phase of PC_PTFE_/ABS, but clearly less in pure PC_PTFE_. The reason for this difference, in terms of the mass loss rate monitored by thermogravimetry, is a shift in the decomposition temperature of PC in the case of PC_PTFE_/ABS towards the decomposition temperature of BDP. 

It is concluded and emphasized that a detailed description of the pyrolysis of each system is the key to understanding the mechanisms and structure-property relationships. Of course, strictly speaking, this contrasts with most of the empirical rules of thumb applied, since these are worked out by averaging the results of many different systems. For instance, emphasizing the specific interactions between flame retardant and pyrolysing polymer no longer supports the point of view that there is a kind of general nitrogen-phosphorus synergism. On the contrary, it suggests that the decomposition of structures containing hetero-atoms like nitrogen or oxygen provides greater opportunity for specific interactions with phosphorus-containing flame retardants than does the decomposition of pure hydrocarbons. Hence, while nitrogenic compounds are an important part of this general picture, strictly speaking there is no P-N mechanism as indicated by the rule of thumb.

As long as the burning of a polymer is characterized by a stable flame above the surface, the pyrolysis occurs under anaerobic conditions [37,38]. Thus, most of the protection goals in distinct fire tests, such as restricted HRR in the cone calorimeter or self-extinction in UL 94, can be achieved by reducing the fuel production or increasing the char yield, respectively, of the anaerobic thermal decomposition of the material. The role of thermo-oxidative processes becomes more important before ignition, after flame-out, or for small and flashing flames. This is often obvious for all of the materials showing afterglow and efficient intumescent systems that avoid sustained flaming. Further, charring during fire, which can be used to reduce the fire risks, must not be confused with the thermal stability monitored in thermal analysis. Surprisingly, merely increasing the thermal decomposition temperature is not a promising fire retardancy approach, since the energy impact during a fire or fire test, respectively, is usually still high enough to reach the decomposition temperature. Indeed, a lot of successfully fire retarded systems start to decompose at lower temperatures than the corresponding pure polymers. This also results in flame retarded systems often igniting earlier than corresponding non-flame retarded polymers. The two-step or multi-step decomposition of materials seems to be the more important general feature of charring systems. However, the two-step decomposition of poly(benzimidazole) (PBI) works as effective intrinsic flame retardancy, whereas the two-step decomposition of blends, such as PC/ABS (ratio = ~5:1), does not. The reason for this is not that some decomposition steps produce fuel and others do not. On the contrary, the key to a successful charring approach is divided into two aspects: first, the characteristic pyrolysis temperature of the pyrolysis front running through the sample corresponds to only part of the thermal decomposition and thus results in remaining residue. In the case of PBI, the pyrolysis temperature corresponds to the first main decomposition step; in the case of PC/ABS it corresponds to the PC decomposition, which is the second main decomposition step. Second, the remaining residue is not subjected to conditions in which the further potential decomposition can build up a pyrolysis front which produces enough fuel for a sustained flame. 

**Figure 1:**
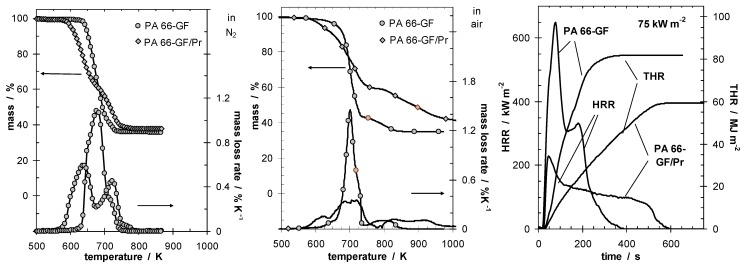
Thermal (left) and thermo-oxidative (middle) decomposition of PA 66-GF and PA 66-GF/Pr (thermogravimetry); cone calorimeter results for HRR and total heat release (THR) (right).

These conclusions are well illustrated by the investigations on glass fiber reinforced polyamide 66 flame retarded with red phosphorus (PA 66-GF/Pr), which demonstrate these charring characteristics [4]. Figure 1 shows the thermal and thermo-oxidative decomposition of PA 66-GF/Pr in comparison to PA 66-GF, as well as the performance in cone calorimeter experiments. For both materials, decomposition is characterized by at least three different processes, which strongly overlap for PA 66-GF and are clearly separated for PA 66-GF/Pr. Some decomposition processes are shifted to lower temperatures, so that the decomposition region is broadened. There is only a small increase in thermal stability for the final decomposition step. Thermal decomposition changes from a one-step decomposition to a two-step decomposition characteristic. In fire tests, PA 66-GF/Pr is an effective charring material, achieving a clear reduction in THE and HRR in the cone calorimeter, as well as the highest self-extinction classification V-0 in the UL 94, whereas in the case of PA 66-GF all of the polymeric material is consumed so that only the glass fibers remain. Thermo-oxidative decomposition of PA 66 was concluded to occur in cone calorimeter experiments before ignition, when a black skin is built up, and during afterglow after flame-out, when a further decrease in mass occurs accompanied by CO production. During the forced-flaming between ignition and flame-out, a stable flame rules out a major influence of oxygen on the decomposition during pyrolysis. 

The mass loss after flaming combustion and the burning time are used to estimate an average effective pyrolysis temperature [4]. This temperature was estimated by the necessary equivalent isothermal thermogravimetry with the same mass loss in the burning time. This is a very rough estimation, of course, since the sample in the cone calorimeter, which is characterized by a temperature profile developing over time, is described by a constant temperature independent of place and time. However, since the specimens investigated were rather thin (2.8 mm) and contained inert filler, and because the fire residue was rather homogenous, the values summarized in Table 3 reasonably estimate the effect. The pyrolysis temperature for PA 66-GF is controlled by the decomposition temperature of the polymer and remains more or less constant for all irradiations used. The calculated temperature is higher than—but still close to—the temperature characteristic for the maximum mass loss rate in thermogravimetry, and the temperature increases slightly with increasing irradiation. The PA 66 is consumed nearly completely by the pyrolysis zone running through the sample. The approximated pyrolysis temperature of PA 66-GF/Pr is characterized by the decomposition temperature of the first decomposition step and thus crucially lower than the temperatures concluded for PA 66-GF. Further, especially for low irradiations, it remains below the temperatures typical for the second decomposition step, so that the residue of the first decomposition functions as stable char in the fire. That the char is, strictly speaking, not thermally stable becomes obvious in this example when irradiation is increased. The resulting surface temperature increases so that more and more polymeric material is consumed by the second decomposition process as well.

**Table 3:** Equivalent decomposition temperatures for PA 66-GF and PA 66-GF/Pr for forced-flaming combustion in the cone calorimeter at various irradiations. The equivalent decomposition temperature is the temperature of an isothermal thermogravimetric experiment resulting in the same mass loss.

	PA 66-GF				
Irradiation/kW m^–2^	30	40	50	60	75
T(equivalent isothermal pyrolysis)/K	693	706	711	723	718
	PA 66-GF/Pr				
Irradiation/kW m^–2^	30	40	50	60	75
T(equivalent isothermal pyrolysis)/K	643	667	684	699	708

As a rule, carbonaceous charring constitutes more than fixing part of the polymer in the condensed phase or diluting the polymeric material. Charring due to the polymer structure entails fixing fuel in the condensed phase, and hence the formation of inorganic residue should always be distinguished from the charring of hydrocarbons. The additional power of charring becomes clear when results using a bomb calorimeter and PCFC for different PC_PTFE_/ABS systems (Table 4) are compared [39].

The bomb calorimeter delivers the heat of combustion per mass for the complete oxidation of a material (h_cBomb_). The PCFC determines the heat of combustion per sample mass for the complete oxidation of the volatiles (h_cPCFC_), whereas the char formed during anaerobic decomposition is not oxidized. Subtracting the PCFC from the bomb calorimeter results (h_cBomb_ – h_cPCFC_) delivers the heat of combustion held in the condensed phase by charring. This value is around a third of the total heat of combustion for PC_PTFE_/ABS (ratio = ~5:1), but the char yield (μ) is only around 0.2, which amounts to one fifth. The heat of combustion held in the condensed phase is zero for inert filler or an inorganic residue. Inert fillers and inorganic residue are accompanied by a reduction in heat release only to the extent that polymeric mass per volume is replaced, but does not cause a reduction by storing the fire load in the residue. The heat of combustion per unit mass of residue (h_c_(residue)) was calculated to lie between 40.9 and 55.7 kJ g^–1^ for the investigated blends (Table 4). When the contributions of inorganic components, such as zinc borate or the inert talc, respectively, were subtracted, the values were similar, between 50.7 and 55.7 kJ g^–1^. Of course, the difference in mass between residue and carbonaceous char can even be larger, when for instance glass fiber reinforced systems are discussed. The heat of combustion per unit mass of carbonaceous char is clearly higher than the h_cBomb_ of the polymer. The enrichment of carbon in the residue becomes apparent, resulting in a carbonaceous, graphite-like or fuel character. Indeed, the h_c_(volatiles) (between 26.1 and 27.4 kJ g^–1^) is correspondingly lower than the h_cBomb_ of the PC_PTFE_/ABS. The favored storage of carbon in the char goes along with a relative increase in the release of less combustible products, such as CO_2_, as was also observed in the evolved gas analysis coupled with thermogravimetry [18]. This result also corresponds with investigations of the chemical composition of char reported in the literature [40,41], demonstrating 90% carbon for PC char. As a consequence, the increased charring of polymers containing hetero-atoms often results in a slight decrease in the effective heat of combustion of the fuel through corresponding fuel dilution, and, if the char is oxidized, also in a significant increase in products such as CO. Neither effect should be misinterpreted as flame inhibition. 

**Table 4:** Comparison between h_cPCFC_ (±0.3 kJg^–1^) from PCFC and h_cBomb_ (±0.03 kJg^–1^) from bomb calorimeter. h_c_(residue) and h_c_(volatiles) are calculated using μ (±0.01) measured in the PCFC; RDP = resorcinol bis(diphenyl phosphate).

	h_cBomb_	h_cPCFC_	h_cBomb_ – h_cPCFC_	μ	h_c_(residue)	h_c_(volatiles)
	kJg^–1^	kJg^–1^	kJg^–1^	gg^–1^	kJg^–1^	kJg^–1^
PC_PTFE_/ABS (ratio = ~5:1)	32.56	22.4	10.2	0.18	55.7	27.3
+ RDP	31.80	20.9	10.9	0.20	54.0	26.2
+ BDP	32.13	21.4	10.7	0.21	51.9	27.0
+ BDP+talc (5%)	30.46	19.9	10.6	0.26	40.9	26.9
+ BDP+zinc borate(5%)	30.47	19.9	10.6	0.25	41.9	26.3

**Figure 2:**
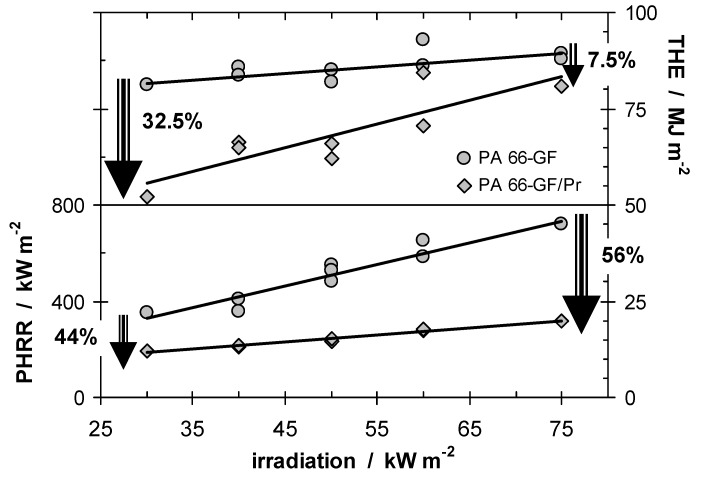
THE and PHRR of PA 66-GF and PA 66-GF/Pr plotted against irradiation. With increasing irradiation, the flame retardancy effect vanishes with respect to THE and increases with respect to PHRR.

Char or inert residue influences fire behavior through two quite different mechanisms. As discussed above, char especially, but also inorganic fillers that replace polymeric material per volume, results in a reduction of the total fuel release and hence THE. In a first approximation, the increase in carbonaceous char yield also yields a proportional decrease in HRR. Further, every fire residue also works as a kind of barrier for heat or mass transfer, suppressing the HRR in particular. Although the same char is the origin of both effects, these mechanisms must be considered as to some degree independent. Again, this conclusion is illustrated exemplarily by the investigations on PA 66-GF/Pr [4,42]. Figure 2 summarises THE and peak HRR (PHRR) for PA 66-GF/Pr in comparison to PA 66-GF.

It is striking that the investigated system shows such a clear differentiation between the behavior of PHRR and THE when the external heat flux is varied. The flame retardancy effect with respect to the THE decreases with increasing irradiation since less char remained, whereas the relative flame retardancy effect with respect to the PHRR increases. The latter clearly indicates the predominant influence of the barrier effect on the HRR. This picture of two different mechanisms being relevant for phosphorus-containing flame retardants acting in the condensed phase is also consistent with the results for systems in which small amounts (<5–15 wt.-%) of inert fillers are added [43,44,45,46]. Such systems show a limited reduction in THE, but often a clearly stronger effect on the PHRR with increasing irradiation due to the surface protection layer.

**Figure 3:**
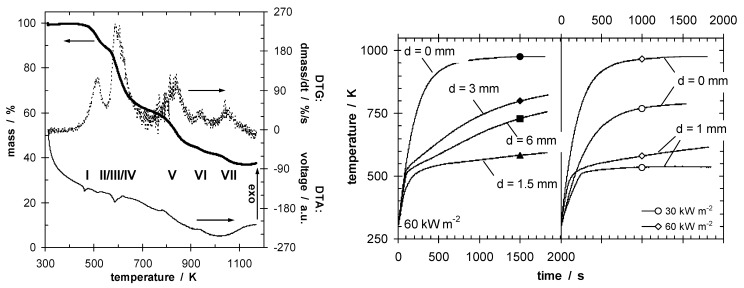
Decomposition (mass, mass loss rate and DTA signal) of an intumescent coating (on left), indicating endothermic char-forming decomposition steps at below 800 K. Temperature at the back of steel plates (right) protected by intumescent coating plotted against time, for different intumescent coating thicknesses (d) and irradiation.

Shielding effects are observed [47,48], especially in the case of effective insulation accompanied by increased re-radiation due to high surface temperatures. Indeed, optimizing the barrier properties so that the HRR drops below a critical value characteristic for passing a test, or even reducing the HRR to cause self-extinction, is a self-contained flame retardancy approach. Most successful is intumescence, where applied heat results in expanding—often multicellular—char insulating underlying material (Figure 3) [5,6,7,49,50]. 

Recent reviews of this concept and its use in polymers, and precise descriptions of the chemistry involved have been published [5,6,7,51,52,53,54,55,56,57,58,59,60]. Indeed, compounds containing phosphorus that increase char formation are often key ingredients in intumescent systems. Temperature differences of up to several hundred degrees Kelvin are observed (Figure 3) [61,62]. Intumescence in polymeric materials can be used to reduce HRR by slowing down the pyrolysis front velocity, or even to achieve extinction before the pyrolysis front passes through the whole specimen. In the latter case the insulation of the char decreases the temperature in the underlying material to keep it from reaching the decomposition temperature of the polymer [23].

### 2.2. Different Phosphorus Flame Retardants in the Same Polymer

**Figure 4:**
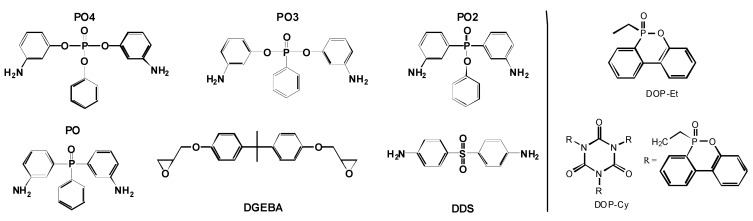
Chemical structures of epoxy and hardeners used (left) and added 9,10-dihydro-9-oxy-10-phosphaphenanthrene-10-oxide (DOPO)-based flame retardants (right).

The search for the best flame retardant to enhance a polymeric material, with respect to applications like electrical engineering and transportation, is currently a typical task in industrial research and development of polymers. Therefore two of the following described examples are close to application. First, the use of reactive and additive flame retardants containing phosphorus is studied in 60 vol.-% carbon fiber reinforced epoxy resin (EP-CF) based on the bifunctional diglycidyl ether of bisphenol A (DGEBA) and different hardeners containing phosphorus (Figure 4) [17,20]. The flame retardants investigated are clearly different in their chemical structure. For all materials, the phosphorus content in the EP was adjusted to around 2.6 wt.-%, amounting to around 1 wt.-% in the EP-CF. The results are compared to materials using 4,4'-diaminodiphenylsulphone (DDS) as a hardener. Second, three different additive flame retardants are investigated in PC_PTFE_/ABS, all of which are based on the same aryl phosphate structure [18].

**Figure 5:**
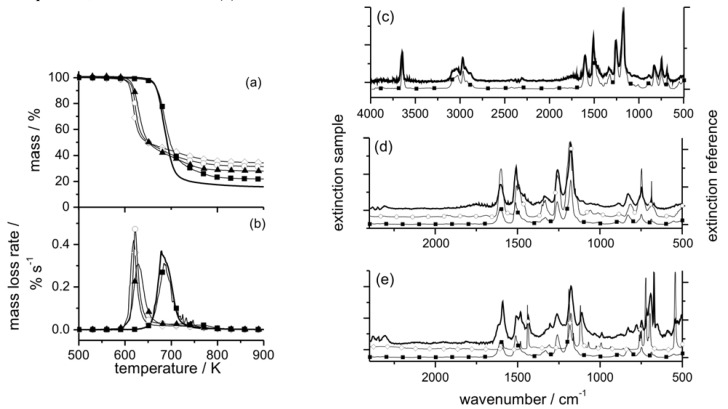
Mass (a) and mass loss rate (b) of EP (thick solid line), EP/PO (filled squares), EP/PO2 (filled triangles), EP/PO3 (open circles), and EP/PO4 (open rhombi) during thermal decomposition (heating rate = 10 K min^–1^). Characteristic evolved gas FTIR spectra (thick solid line) of EP/PO at 40 min (c, d) and 48 min (e) with reference spectra from a database (filled squares = bisphenol A, open circles = phenol, open rhombi = triphenylphosphine oxide). Remaining bands ~1745 cm^–1^ (c, d) are attributed to carbonyl compounds, and at 673 cm^–1^ (e) to benzene.

**Figure 6:**
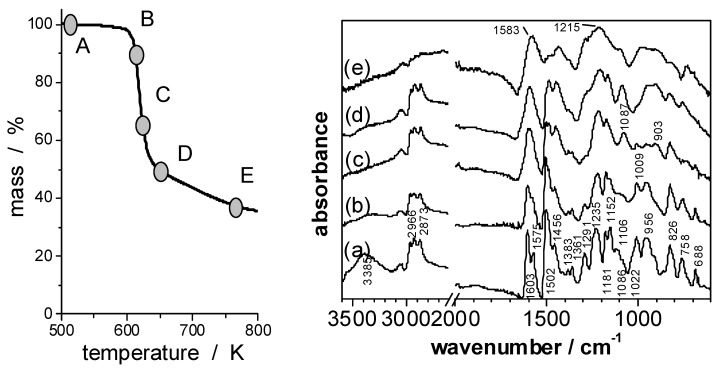
FTIR spectra of EP/PO4 (A, a) and of its solid pyrolysis products obtained under N_2_ by heating to 10% weight loss (B, b); 35% weight loss (C, c); 53% weight loss (D, d) and to 768 K (E, e).

Recently several overviews have been published on the different flame retardancy approaches proposed, thermal decomposition, fire behavior and recent developments [63,64,65] in which phosphorus based systems have been emphasized as suitable halogen-free alternatives. Phosphate, phosphine oxide and phosphonate structures incorporated in epoxy or hardener have been reported [66,67,68,69,70,71]. In order to investigate the influence of the “oxidation state” of the phosphorus on fire retardancy, the pyrolysis of epoxy resins containing phosphine oxide (PO), phosphinate (PO2), phosphonate (PO3), and phosphate (PO4) were investigated comprehensively (Figure 5, Figure 6) [17,20].

The initial decomposition of EP/PO2, EP/PO3, and EP/PO4 is determined decisively by incorporating the P–O–C_arom_ structure into the side group (EP/PO2) or P–O–C_arom_ into the chain structure (EP/PO3 and EP/PO4) instead of the more stable P–C_arom_ link. The initial scission of the hardeners is accompanied and followed by NH_2_ reacting with the –OH group of the epoxy network, as shown in the FTIR spectra of the solid products *versus* conversion (Figure 6). A decrease in thermal stability through incorporating phosphate and phosphonate in EP was also reported by other groups [72,73,74,75]. The main decomposition step of EP/PO is determined by the decomposition of the epoxy structure as reported in the literature [67,76,77]. However, an additional subsequent decomposition and an increase in char were observed. Main decomposition pathways are proposed based on a comprehensive characterization of thermal decomposition (TG, kinetics), the volatile decomposition products sketched in Figure 5 for EP/PO and the remaining residue (TG-FTIR, FTIR-ATR and XPS) [20]. Reactions during decomposition that result in the formation of P–N are presumed, since they seem to be reasonable from FTIR, XPS, and in comparison to the hardener decomposition. However, the key reaction in the epoxy resins is the initial reaction between P–O–C_arom_ and –OH, forming P–OH. Of course, this amounts to the formation of phosphorus-containing acids. This initial reaction destroys the EP network and thus determines the decomposition temperature range, activation energy and mass loss. 

The phosphorus-containing flame retardants investigated showed a variety of influences on the decomposition of EP. They influenced char formation in the condensed phase, the release of phosphorus-containing products into the gas phase, and changes in pyrolysis characteristics like decomposition temperature and the number of separate decomposition steps (Figure 5). Significant interactions were observed between the epoxy structure and the groups containing phosphorus, clearly altering the decomposition pathway. The main results for the EP based on the systematic investigation of various hardeners containing phosphorus are summarized as follows:

➢ Phosphorus-containing groups alter the decomposition of the EP, resulting in a multi-step decomposition. Mass losses between around 15 and 20 wt.-% occur for the decomposition subsequent to the main decomposition step. The high temperature residue is increased.
➢ Phosphorus-phenoxy groups incorporated in the EP structure decrease the decomposition temperature, enhance water elimination, and reduce mass loss during the main decomposition step.
➢ Phosphorus-containing acids are formed and enhance charring. Apart from carbonaceous char, PxOy and PxNyOz occur in the high-temperature residue. The condensed phase effects are strongest for phosphate and decrease with the decreasing oxidation state of the phosphorus. They are of minor importance for phosphine oxide.
➢ Volatile decomposition products containing phosphorus are released into the gas phase. The effect is the strongest for phosphine oxide and decreases with the increasing oxidation state of the phosphorus. It vanishes for phosphate.

The fire behavior (Table 5) of corresponding carbon fiber reinforced composites (EP-CF, EP-CF/PO, EP-CF/PO2, EP-CF/PO3, EP-CF/PO4) was studied. The flammability and the fire behavior for forced flaming conditions were investigated by means of limiting oxygen index (LOI), UL 94 tests and a cone calorimeter used with different irradiations [20]. The flame retardancy mechanisms were discussed for the gas and condensed phases. The fire behavior of the modified EP was compared to non-fire-retarded EP cured with DDS as a hardener. It turned out that this comparison is not perfectly systematic, as the -SO_2_- group, in particular, increases the intrinsic fire retardancy of the polymeric structure in fire performance. However, the DGEBA-DDS system (EP-CF) serves as an interesting reference value for an established system. The main results based on systematic investigation of various hardeners containing phosphorus are summarized as follows:

➢ Flame inhibition is observed. The effect is strongest for phosphine oxide and decreases with the increasing oxidation state of the phosphorus. It vanishes for phosphate.
➢ Reduced thermal stability results in a decreased time to ignition (t_ig_)
➢ No significant effect from changing the decomposition with respect to the size of the mass loss step or multi-step decomposition
➢ No significant effect from additional charring
➢ Flammability is ordered: EP-CF/PO < EP-CF/PO3 ≤ EP-CF/PO2 < EP-CF ≤ EP-CF/PO4.
➢ Fire risks like PHRR in developing fire are ordered: EP-CF/PO < EP-CF/PO2 ≤ EP-CF < EP-CF/PO3 < EP-CF/PO4.

**Table 5:** Propensity for phosphorus release and charring determined for the EP by thermal analysis, combustion efficiency multiplied by the effective heat of combustion (χ·h_C_), PHRR and THE determined by cone calorimeter and flammability (LOI, UL 94) for the composites (60 vol.-% carbon fibers); phosphorus content in the EP-CF is around 1 wt.-% (with respect to the EP = ~2.6 wt.-%).

	From TG		Cone calorimeter			Flammability	
EP-CF with	P-release	Charring	χh_C_ °	PHRR ^#^	THE °	LOI	UL94
			kJ g^–1^	kW m^–2^	MJ m^–2^	%	
			± 1	± 40	± 0.5	± 1	
DDS *			22	375	24.4	31	HB
PO	+++	+	17	237	24.0	39	V-1
PO2	+	++	21	366	21.3	34	HB
PO3	++	+++	20	536	23.8	36	HB
PO4		++++	22	623	24.1	29	HB

^#^ value for 50 kW m^–2^ irradiation ° average over all measurements for different irradiations * DDS = benchmark, not a systematic comparison

The composite containing phosphine oxide incorporated in the polymeric structure (EP-CF/PO) shows superior performance in terms of flammability (UL 94 and LOI) and fire behavior (cone calorimeter), because, in the case of the investigated systems, it yields the most efficient release of phosphorus in the gas phase. In contrast, the phosphate incorporated as a reactive flame retardant into the epoxy-based composite (EP-CF/PO4) does not display any significant release of phosphorus into the gas phase. The flammability and fire behavior of the carbon fiber (60 vol.-%) reinforced epoxy composites are influenced by condensed phase and gas phase flame retardancy mechanisms, but flame inhibition plays the far more important role. This result may be a crucial conclusion for composites consisting of thermosets with high filler content, in which the relative charring impact is small, and some degree of thermo-oxidative char decomposition probably occurs from the back of the specimen during burning.

Using the general conclusions of this systematic study, some promising routes are proposed for the tailored flame retardancy of EP-CF:

➢ Using compounds that act as precursors for the release of phosphorus-containing volatiles
➢ Enhancing the release of phosphorus-containing volatiles by using an additive approach or "side group-like" incorporation into the polymer structure.

These routes are pursued using 10-ethyl-9,10-dihydro-9-oxa-10-phosphaphenanthrene 10-oxide (DOP-Et) or 1,3,5-tris[2-(9,10-dihydro-9-oxa-10-phosphaphenanthrene 10-oxide-10-)ethyl]1,3,5-triazine-2,4,6(1H,3H,5H)-trione (DOP-Cy) as additives (Figure 4) based on the DOPO structure, which is believed to release PO efficiently, at least in a number of polymeric systems [78].

**Figure 7:**
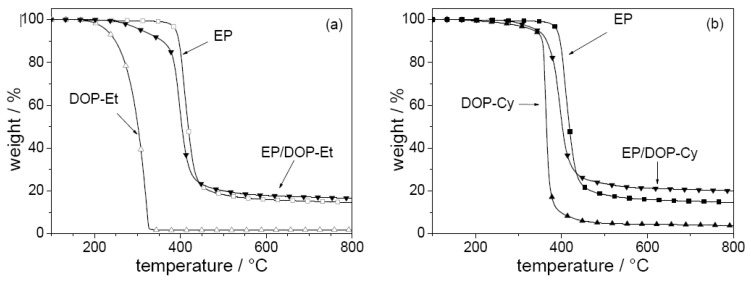
Mass (thermogravimetry under nitrogen; heating rate = 10 Kmin^-1^) of a) EP, DOP-Et, and EP/DOP-Et and b) EP, DOP-Cy, and EP/DOP-Cy.

The pyrolysis and fire behavior are investigated for EP and corresponding EP-CF, both flame retarded with the additives DOP-Et and DOP-Cy [17]. Both flame retardants show an interaction in the condensed phase influencing the decomposition of EP (Figure 7). The decomposition pathways of the different materials were investigated in detail by examining the mass loss, the decomposition temperatures, the evolved gases, and the chemical composition of the residues for different conversions and temperatures. The onset and maximum decomposition temperatures are decreased, the char yield is increased, and phosphorous volatiles are released. The decreased decomposition temperature results in a decreased ignition resistance of EP-CF. The increased char yield and the release of compounds containing phosphorus significantly improve the flammability and fire properties of EP-CF as demonstrated by means of LOI, UL 94, and cone calorimeter, respectively (Table 6). For both flame retardants two main mechanisms affect fire behavior: char enhancement and flame inhibition. DOP-Et shows the more efficient release of phosphorus compounds, but DOP-Cy the higher charring. The fire properties achieved, and especially the V-0 classification, are interesting with respect to industrial exploration. The V-1 (EP-CF/DOP-Et) and the V-0 (EP-CF/DOP-Cy) classification correspond to very large LOI values of 44% and 49%. Also, the V-1 classification of EP-CF/PO discussed above was achieved for a very high LOI value of 39%, whereas the EP-CF/PO3 shows an LOI of 36% but only an HB classification. That such high LOI values go along with achieving V-0 classification has been reported before [79] and seems to be confirmed as a general result of this work for such composites. The use of DOPO-based flame retardants results in the release of most probably PO into the gas phase, and in phosphorus remaining in the condensed phase during pyrolysis. The release of PO results in efficient flame inhibition, whereas phosphorus in the condensed phase enhances char formation due to its interaction with EP during pyrolysis. Tailored DOPO-based flame retardants provide efficient flame retardancy at very low P content, in the order of 2 wt.-% with respect to EP, or in the order of 1 wt.-% with respect to EP-CF, respectively. In general, DOPO-based flame retardants show a high propensity to achieve efficient flame inhibition, since the DOPO structure acts as a precursor for the release of PO during pyrolysis. Hence, DOPO-based flame retardants are especially suitable for use in materials and protection goals that favor a gas phase mechanism, such as flammability tests for thermosets with large amounts of filler.

**Table 6:** Fire behavior of EP-CF, EP-CF/DOP-Et, and EP-CF/DOP-Cy.

	Thermal analysis		Cone calorimeter			Flammability	
EP-CF with	P-release	Charring	χh_C_ °	PHRR ^#^	THE °	LOI	UL94
			kJ g^–1^	kW m^–2^	MJ m^–2^	%	
			± 1	± 40	± 0.5	± 1	
EP-CF			22	375	24.4	31	HB
EP-CF/DOP-Et	+++	+	16	226	15.4	44	V-1
EP-CF/DOP-Cy	+++	+	17	252	17.0	49	V-0

# for q_ex_ = 50 kW m^–2^ ° average over all measurements for different irradiations

However, it also becomes clear that kind and intensity of the flame retardancy effect depends, not only on the chemical structure of the DOPO compounds, but also on the interactions with the polymer during thermal decomposition. Significant differences in the flame retardancy mechanism and efficiency are achieved by using different DOPO-based flame retardants with the same phosphorus content. Indeed, the investigation demonstrates not only the potential of the DOPO structure for phosphorus release and resulting flame inhibition, but also its potential for the combination of flame inhibition and charring, as well as a probable potential for charring itself as a main mechanism.

So far, the results for the systematic variation in the structure of the flame retardants indicate structure-property relationships, which raise the question as to how specific they are with respect to the investigated polymeric material. Superior flame retardancy was reported for phosphine oxide or phosphates, respectively, depending on the systems investigated and due to the different mechanisms occurring in the gas and condensed phases. The fire retardancy mechanisms and efficiency are sensitive for the decomposition, controlling char yield and phosphorus release. The chemical structure of flame retardants such as phosphine oxide and phosphonate and how they are incorporated (additively or reactively) are the main approaches to optimize polymeric materials. However, these approaches are only a means to an end with respect to the causal mechanistic structure-property relationships. The chemical structure merely serves as a prerequisite for the decomposition and release, which determine flame retardancy mechanisms and efficiency. To illustrate this, the aryl phosphate-based flame retardants triphenyl phosphate (TPP), RDP and BDP, are compared in PC_PTFE_/ABS (ratio = ~5:1). They are very similar in their chemical structure, but different in their volatility. Levchik and Weil [80,81] recently gave several overviews of flame retardants for PC and PC/ABS, in which phosphorus-based systems have been emphasized as appropriate halogen-free alternatives in electronic engineering.

Levchik *et al.* [82,83] examined the fire behavior of PC/ABS blends fire retarded with three different aryl phosphates: TPP, RDP and BDP. They postulated that the primary fire retardant action of BDP probably takes place in the condensed phase. According to Lyons [84], phosphorus compounds act like acid precursors, and acids participate in char formation through esterification and dehydration. On the other hand, it has been described in the literature that flame retardants containing phosphorus, such as phosphate esters, can act in the gas phase as well as through flame inhibition [11,26].

Hence, the thermal decomposition (TG, TG-FTIR, kinetics and FTIR-ATR), the flammability (LOI, UL 94) and the fire behavior (cone calorimeter) (Table 7) of aryl phosphates in PC_PTFE_/ABS material, were investigated [18].

**Table 7:** Propensity for phosphorus release and charring determined by thermal analysis for PC_PTFE_/ABS (ratio = ~5:1) materials, combustion efficiency multiplied by the effective heat of combustion (χ·h_C_), PHRR and THE determined by cone calorimeter and flammability (LOI, UL 94).

	Thermal analysis		Cone calorimeter			Flammability	
	P-release	Charring	χh_C_ °	PHRR ^#^	THE°	LOI	UL94
			kJ g^–1^	kW m^–2^	MJ m^–2^	%	
			± 0,5	± 25	± 1.5	± 1.0	
PC_PTFE_/ABS			23.6	555	55.7	23.6	HB
+ BDP	**++**	**++**	19.9	357	45.4	28.1	V-0
+ RDP	**+++**	**+**	18.7	373	44.3	29.8	V-0
+ TPP	**+++**		18.6	377	47.4	29.8	V-0

^#^ value for 50 kW m^–2^ irradiation ° average over all measurements for different irradiations

TPP is released almost completely before the decomposition of ABS and PC. RDP and BDP induce additional charring of PC, indicated by increased residue and shifting the PC decomposition. The flame retardant mechanisms were proposed based on the results of various methods, in particular the processes and interactions during pyrolysis that result in gas phase and condensed phase flame retardancy mechanisms. All aryl phosphates investigated act through flame inhibition in the gas phase, whereby TPP and RDP perform slightly better than BDP. Further, RDP shows some, and BDP a marked, additional condensed phase action. They increase the charring of PC during decomposition. The residue is increased, accompanied by a shift in the main decomposition of PC to higher temperature. This effect is the largest when adding BDP, whereas for TPP no significant condensed phase action was observed at all. TPP shows only a gas phase action, since it vaporized at temperatures below PC decomposition in PC_PTFE_/ABS. The dependency of the condensed phase activity of the aryl phosphates on decomposition temperature areas corresponds to the difference of BDP action in pure PC_PTFE_ *versus* PC_PTFE_/ABS discussed above. The resulting flammability (LOI, UL 94) of PC_PTFE_/ABS + BDP, PC_PTFE_/ABS + RDP, and PC_PTFE_/ABS + TPP is rather similar; RDP and TPP show a slightly better LOI value. The combination of gas phase and condensed phase actions of BDP results in a slightly superior performance in fire behavior under forced-flaming conditions, particularly with respect to fire load and decreasing flame spread.

Comparing the three aryl phosphates in PC_PTFE_/ABS reveals that minor changes in the chemical structure can result in changes in interactions during pyrolysis and in the resulting fire retardancy mechanisms and efficiencies.

### 2.3. Phosphorus Flame Retardants in Different Polymers

The results of the previous section already indicate that the fire retardancy mechanisms caused by each flame retardant are determined not only by its structure, but also by the interaction with its chemical surroundings during pyrolysis. Hence, it was concluded that the structure-property relationships are specific with respect to the polymer investigated. As a consequence, not only do different flame retardants work differently in a polymer, but a single flame retardant also may work differently in different polymers. In order to illustrate and discuss this aspect, the same flame retardant is investigated using varying polymeric matrices.

As an example 6-10 wt.-% Pr was investigated, in polymer structures based on hydrocarbons (high impact polystyrene, HIPS), glass fiber reinforced polyester containing oxygen (PBT-GF), and PA 66-GF containing oxygen and nitrogen in their backbone [4,13,85,86]. Pr is always encapsulated for flame retardancy to rule out any reaction with atmospheric moisture to form phosphine. Pr is an effective flame retardant in a wide range of polymeric materials [12,87,88,89,90], in particular for polymers containing oxygen in their main chain. It was reported to induce char formation in polymers containing nitrogen or oxygen in their main chain [91,92], but also to work in the gas phase for hydrocarbon polymers [93].

The thermal decomposition of PA 66-GF with and without Pr was reported in detail in previous sections (Figure 1), and corresponds to comparable studies and reviews [94,95,96,97,98]. The change from a one-step into a two-step decomposition goes along with a change in pyrolysis product and decomposition activation energies *versus* conversion [4]. Indeed, previous studies reported that the change in kinetics of the decomposition plays a major role in the system [35,99]. For PA 66 the main decomposition products of the first two processes were cyclopentanone (TG-FTIR: 1,764 cm^–1^, 2,978 cm^–1^) carbon dioxide (CO2) (TG-FTIR: 2,360 cm^–1^, 2,335 cm^–1^) ammonia (TG-FTIR: characteristic patterns in particular between 800 and 1,200 cm^–1^), hydrocarbon and amine species (TG-FTIR: 2,924 and 3,017 cm^–1^). The third process was accompanied by hydrocarbons and amine species. For PA 66-GF/Pr, the decomposition processes were quite similar, but the evolution of hydrocarbons and amine species was reduced for the first two processes. It was concluded that Pr changes the decomposition temperature and pathway due to specific interactions. The resulting processes were assumed to be influenced also by the pH value, the presence of water, and the presence of Pr, which is oxidized in the condensed phase under such conditions [100]. It should be noted that the PA 66-GF investigated contains <2 wt.-% additional additives used as processing and stabilization agents, which are assumed to enhance the condensed phase action of Pr and the proposed change in the decomposition pathway. Even though a detailed study on this aspect was not performed, it is reasonable to assume such an enhancement when the results are compared to those reported for comparable systems [91,98,101].

The pyrolysis of PS in HIPS and in HIPS/Pr was characterized by a single main decomposition step [13,86]. The release products are dominated by monomers (TG-FTIR: 3,070 cm^–1^; TG-MS: 104, 103, 78, 77, 51, 50 m/e), methylstyrol (TG-MS: 118, 116, 115, 58 m/e) and dimers and trimers of PS (TG-FTIR: 2,930 cm^–1^). The results for the thermal decomposition of HIPS corresponded to those of comparable studies [102,103,104]. HIPS/Pr shows only a minor change in the high-temperature flank of the mass loss rate, indicating an additional mass loss at slightly higher temperatures that correspond to the release of most of the phosphorus as P_4_ (TG-MS: 124 m/e) as proposed by others for comparable systems [93,105]. No residue was formed. Adding Pr did not influence the decomposition process of the polymer significantly. Phosphorus vaporizes independently of the polymer matrix.

PBT-GF and PBT-GF/Pr decomposed very similarly [85]. The characteristic temperatures and evolved gases showed no evidence of a change in decomposition mechanisms. The main decomposition products were butadiene (TG-FTIR: 908 cm^–1^; TG-MS: 54 m/e), terephthalic acid (TG-MS: 166 m/e), benzoic acid (TG-FTIR: 1,762 cm^–1^; TG-MS: 122 m/e), CO_2_ (TG-FTIR: 2,360 cm^–1^; TG-MS: 44 m/e), and in the case of PBT-GF/Pr also P_4_ (TG-MS: 124 m/e). It was concluded that the decomposition consisted of two main pathways based on thermal scission and an ester hydrolysis, as has been reported for comparable PBT compounds [106,107,108,109]. The thermal decomposition of polyesters was reviewed recently [110]. Analogous to Pr in HIPS, under these conditions Pr vaporized independently in PBT-GF. When a small amount of alkali adjuvant such as ZnO was added to PBT (PBT*), similar to the additional additives used in PA 66, a slight change in decomposition mechanisms was observed for PBT*-GF and PBT*-GF/Pr [85]. The onset decomposition temperature of PBT* was shifted to lower values and a second small decomposition step was detected above the main pyrolysis. The products of thermal scission, butadiene and terephthalic acid, were still detected, but with less intensive acid signals. Benzoic acid was not observed, but tetrahydrofuran (THF) (TG-FTIR: 2,980 cm^–1^; TG-MS: 72, 71, 42, 41 m/e) was, and for subsequent higher temperatures also benzene (TG-FTIR: 670 cm^–1^; TG-MS: 50, 51, 77, 78 m/e). This behavior corresponded to the alkaline-catalyzed ester hydrolysis process. Vaporized phosphorus was still detected, but Pr was now partly oxidized in the condensed phase.

In conclusion, during the pyrolysis of PA 66-GF/Pr, phosphorus remains mainly in the condensed phase, whereas it is released completely and nearly completely in HIPS and PBT-GF, respectively. No significant charring of the polymer is expected for HIPS/Pr and PBT-GF/Pr, respectively, whereas PA 66-GF/Pr shows a clear change to a two-step decomposition of the polymer, which harbors the potential for efficient charring. Some charring is expected when PBT is replaced by PTB*.

PA 66-GF without Pr showed typical fire behavior for non-charring polymers containing inorganic glass fiber as inert filler [111]. When Pr was added, the PA66-GF samples were transformed into char-forming materials, which led to a reduction in THE and a reduction in the PHRR (Figure 1). The mass loss and the THE corresponded to each other, which means the ratio THE/TML = χ**·**hc is constant, indicating the absence of relevant gas phase mechanisms. The char influenced fire behavior via two quite independent mechanisms. First, it acted as a barrier layer restricting the fuel support rate. Second, the thermally stable char decreased the total amount of fuel. 

**Figure 8:**
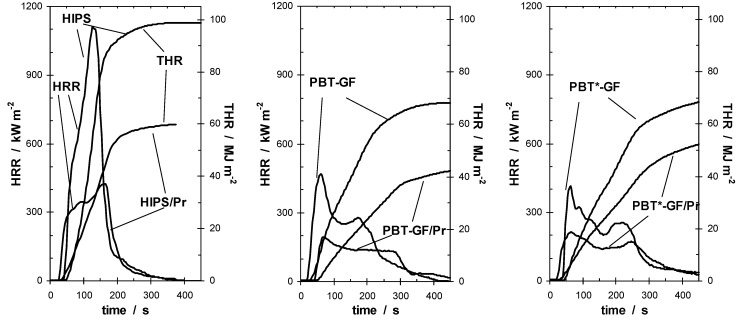
Cone calorimeter results (HRR and THR; irradiance = 50 kW m^–2^) for HIPS and HIPS/Pr (left), PBT-GF and PBT-GF/Pr (middle), PBT*-GF and PBT*-GF/Pr (right). Pr reduces PHRR and THE through flame inhibition.

For all HIPS samples, no charring was observed in the cone calorimeter experiments. The HRR curves of HIPS and HIPS/Pr are typical for non-charring thermoplastic specimens of intermediate thermal thickness. The fire behavior of HIPS/Pr showed a reduction in HRR and THE in comparison to HIPS (Figure 8). The fire retardancy was due to a decreased THE/TML = χ**·**hc. Adding Pr to HIPS resulted in flame inhibition in the gas phase. The combustion behavior of PBT-GF and PBT*-GF was characterized by an intensive PHRR, followed by a zone of steady HRR. Hence the HRR curve was typical for polymers containing inert glass fiber as filler [111] and was quite analogous to PA 66-GF. The materials containing Pr showed a significant reduction in HRR and in THE compared with their corresponding PBT-GF materials (Figure 8). For PBT/Pr, no additional char was formed, whereas for PBT*-GF/Pr small amounts of char remained, especially at low external heat fluxes. Hence, a partial condensed phase action of Pr was indicated for PBT*-GF/Pr. For all materials, phosphorus release resulted in a reduction of the THE/TML based on gas phase mechanisms, which obviously caused the corresponding reductions in PHRR and THE. PBT*-GF/Pr showed a smaller reduction, indicating the partial condensed phase action of Pr. However, the additional char slightly improves performance in terms of flammability behavior (Table 8).

**Table 8:** LOI of PBT-GF materials.

	PBT-GF	PBT-GF/Pr	PBT*-GF	PBT*-GF/Pr
LOI / % (±1 %)	19.6	25.4	21	26.7

In PA 66-GF Pr acted in the condensed phase, in HIPS in the gas phase and in PBT-GF mainly in the gas phase. The activity of phosphorus as a flame retardant in the condensed phase depends on the interaction between the flame retardant and polymer decomposition during pyrolysis, and thus on the polymer structure and its characteristic decomposition pathway, water content, and pH value. Using adjuvants, the different mechanisms can be triggered to some extent. The presence of heteroatoms like oxygen or nitrogen enabled a condensed phase mechanism of Pr only when the resulting decomposition pathway enabled its charring influence. 

**Figure 9:**
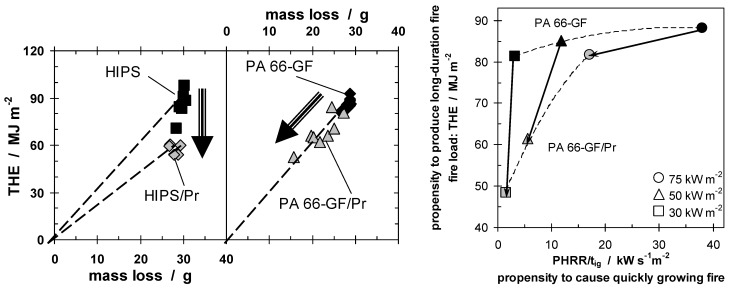
THE plotted against mass loss for HIPS, HIPS/Pr, PA 66-GF, and PA 66-GF/Pr, illustrating the flame inhibition of Pr in HIPS and the charring in PA 66-GF (on left). Fire behavior assessment (right), for PA 66-GF and PA 66-GF/Pr, for different fire scenarios (different heat fluxes). The fire load (THE) and a fire growth index (PHRR/t_ig_), among the two most important fire risks, are plotted on the same graph.

Figure 9 illustrates the fundamental difference in the fire retardancy mechanism of Pr used in HIPS and PA 66-GF. The THE for both materials, with and without Pr as a fire retardant, are plotted against the mass loss. The lack of significant change in the mass of residue shows that phosphorus in HIPS vaporizes nearly completely and no additional charring occurs. The THE is reduced by flame inhibition, reducing the product of the effective heat of combustion of the volatiles and combustion efficiency (χ·hc), which is indicated by the reduced slope. For phosphorus in PA 66-GF χ·hc remains constant, whereas the reduction in THE is caused by a decreased mass loss. This shows that Pr induces char formation in the condensed phase.

Whereas HIPS, HIPS/Pr, and PA 66-GF, show a rather similar THE for the different measurements and applied irradiation, the data for PA 66-GF/Pr show a clear shift with changing irradiation. This accords with the above reported result that not only the PHRR of PA 66-GF/Pr is dependent on the external irradiation applied, but also the THE (Figure 2). It is important to take this behavior into account accurately to assess the fire performance scientifically and comprehensively. Figure 9 uses the Petrella approach in order to assess the fire risks of both PA 66-GF and PA 66-GF/Pr at different external heat fluxes. As the two most important fire risks, the fire load (THE) observed in the cone calorimeter, and a fire growth index (PHRR/t_ig_) rather than a real flame spread, are sketched graphically for PA 66-GF with and without Pr as a flame retardant. The assessment is valuable for different applications, fire scenarios and fire tests, as these correspond to different external heat fluxes and define different demands on fire retardancy in terms of the long duration and growth of a fire. The data for both materials show that, with increasing heat flux, the fire hazards increase in terms of fire growth, as expected, since a higher heat flux results in an increase in fuel production rate. The THE of PA 66-GF is fairly constant, since the polymer is combusted nearly completely for all heat fluxes used, whereas the THE decreases most for the flame retarded material at low heat flux, because the char formation is greatest. This effect diminishes with increasing heat flux. The fire hazard is reduced by the flame retardant, either by reducing the THE or by reducing the fire growth rate. The best fire retardancy would yield reduction in both, represented by approaching the origin. In the case of Pr in PA 66-GF, combustion is complete at the highest heat flux, with or without flame retardant, so the THE is almost the same, but the fire growth index is almost halved. Conversely, at low heat fluxes, not only is the fire growth index reduced, but the THE is almost halved as well. The change in the fire scenario changes the effectiveness of Pr added to PA 66-GF in two of the most important fire properties. Pr in PA 66-GF works best for low external heat flux. Flammability tests like LOI and, much more important, UL 94, are fire scenarios with low external heat flux. PA 66-GF/Pr has been optimized specifically for use as a commercially successful product in electronics and electrical engineering. The dependency of the efficiency on the irradiation and, in particular, the specific impact on different fire hazards is concluded to be characteristic for condensed phase mechanisms of phosphorus flame retardants.

### 2.4. Phosphorus Flame Retardants in Combination with Other Flame Retardants and Synergists

The results of the previous chapter already indicate that even very small amounts of additional additives, like <2 wt.-% metal oxide, can significantly enhance or change the interaction of phosphorus-containing flame retardants with their chemical surroundings during pyrolysis. Hence, it was concluded that the combination of phosphorus flame retardants with other flame retardants, synergists or adjuvants can be used to control or change mechanisms and thus optimize flame retardancy efficiency. Indeed, this is quite a popular approach in developing flame retarded materials based on phosphorus-containing flame retardants to compete with systems containing halogen. Synergisms in flame retardant systems have been reviewed [22,112]. It seems that there is no general synergist for phosphorus flame retardants as is known for flame retardants containing halogen and antimony. However, various metal compounds, borates, inorganic fillers, and flame retardants containing nitrogen and phosphorus are reported to show synergism with phosphorus flame retardants. In some systems the main point is to combine different flame retardancy mechanisms. In other systems chemical reactions lead to other pyrolysis products such as metal phosphates in the condensed phase rather than the volatilization of phosphorus oxides. The char structure is changed and an inorganic glassy coating can occur. In other systems physical effects are exploited, such as a better char strength or insulation due to a changed residue morphology or changed melt flow and dripping properties, However, it should be noted that for hydroxides with ammonium polyphosphate (APP) used in poly(ethylene vinyl acetate) (EVA), for instance, both synergisms and antagonisms are reported [113,114,115,116]; since effects like the formation of water and of metal phosphates actually can increase or decrease flame retardancy efficiency. Also for combinations of APP with layered silicate, both synergism and antagonism are reported, since the change in melt viscosity and char properties can increase or decrease flame retardancy [7,117]. In order to discuss such aspects in detail, some systems are investigated.

**Figure 10:**
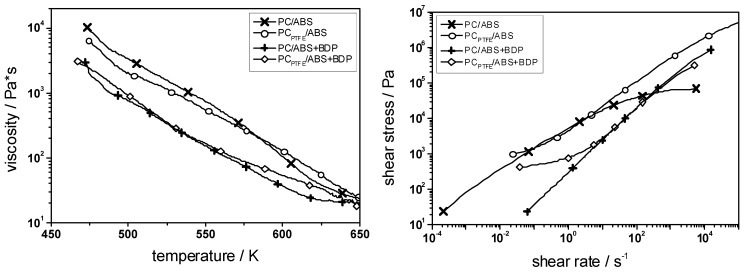
Rheology for PC/ABS, PC_PTFE_/ABS, PC/ABS + BDP and PC_PTFE_/ABS + BDP. BDP works as plasticizer reducing the viscosity; PTFE works as an anti-dripping agent increasing the viscosity towards the decomposition temperature and introducing a flow limit at low shear rates.

PC/ABS blends (ratio = ~5:1) with BDP (12.5 wt.-%), with PTFE (0.45 wt.-%), and with a combination of both were examined with respect to their decomposition, melt viscosity, flammability (reaction to a small flame) and fire behavior (forced-flaming conditions) [18]. The physical and chemical mechanisms and effects of the additives were revealed as well as the synergisms between them. As mentioned above, adding BDP results in flame inhibition in the gas phase and enhanced charring in the condensed phase. However, it is also an efficient plasticizer, reducing the melt viscosity crucially (Figure 10). 

Thus, not only PC/ABS shows self-extinction in the vertical UL 94 test due to burning dripping (V-2), but also PC/ABS/BDP (Table 9). Indeed, it shows a clearly enhanced propensity for dripping. PTFE is a very important real synergist for the effectiveness of aryl phosphates in PC/ABS as it is contained in many thermoplastics. It only moderately changes the viscosity of the polymer melt—in fact, only in the temperature region towards decomposition was a significant increase observed—but clearly introduces a flow limit at low shear rates (Figure 10), which prevents melt flow and dripping, particularly in flammability tests like UL 94 (Table 9). Adding PTFE inhibits dripping in PC_PTFE_/ABS so that self-extinction is no longer observed and HB classification is obtained. Avoiding dripping in combination with aryl phosphates, PC_PTFE_/ABS/BDP achieves V-0, the prerequisite for challenging applications. Only when PTFE stabilizes the pyrolysis zone in the vertical UL 94 test is the flame retardancy action of BDP exploited. Further, thermal analysis clearly proves an additional chemical mode of action of PTFE in the case of PC_PTFE_/ABS + BDP, whereas PTFE has no impact at all on the decomposition of PC/ABS [18]. Adding PTFE enhances the residue and shifts the decomposition temperature of PC to higher temperatures only in PC_PTFE_/ABS/BDP, which are the typical changes also observed in the interaction of BDP with the decomposition of PC in PC/ABS. Adding PTFE enhances the cross-linking of BDP and PC due to esterification. It was proposed that PTFE yields an additional acidic influence during pyrolysis [18]. Thus PTFE acts mainly physically as anti-dripping agent, but also chemically in some systems. The combination of BDP and PTFE in PC_PTFE_/ABS + BDP yields remarkably superior flame retardancy based on synergistic effects. What is more, PTFE enables the fire retardancy action of BDP itself with respect to a V-0 classification in UL 94, and hence gives birth to the industrial exploitation of this system.

**Table 9:** Phosphorus release and charring determined by thermal analysis, combustion efficiency multiplied by effective heat of combustion (χ·h_C_), PHRR and THE determined by cone calorimeter and flammability (LOI, UL 94) for PC/ABS, PC_PTFE_/ABS, PC/ABS + BDP, PC_PTFE_/ABS + BDP.

	Thermal analysis		Cone calorimeter			Flammability	
	P-release	Charring	χh_C_ °	PHRR^#^	THE°	LOI	UL94
			kJ g^–1^	kW m^–2^	MJ m^–2^	%	
			±0.5	±25	±1.5	±1.0	
PC/ABS		**+**	24.0	670	62.6	23.2	V-2
PC_PTFE_/ABS		**+**	23.6	555	55.7	23.6	HB
PC/ABS/BDP	**+++**	**++**	18.7	493	48.8	26.0	V-2
PC_PTFE_/ABS + BDP	**++**	**+++**	19.9	357	45.4	28.1	V-0

^#^ for q_ex_ = 50 kW m^–2^ ° average over all measurements for different irradiations

Apart from the above described adjuvants that enable efficient flame retardancy such as PTFE in PC_PTFE_/ABS + BDP, combinations of flame retardants or of flame retardant and a synergist often are proposed as an alternative to an increase in flame retardant content that would critically disturb the material properties (cost, mechanical properties, color, *etc.*) or yield no further increase in the flame retardancy effect. Indeed, the flame retardancy of phosphorus flame retardants such as Pr was reported to level off with increasing amounts [92,108,118]. This effect is also indicated in Table 1.

**Figure 11:**
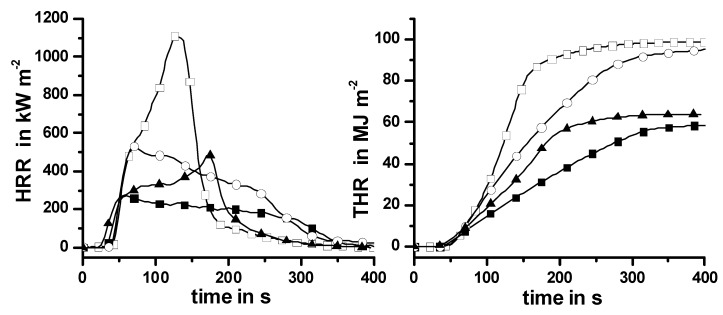
Heat release rate (HRR) and total heat release (THR) of HIPS (open squares), HIPS/Pr (filled triangles), HIPS/ Mg(OH)_2_ (open circles) and HIPS/Pr/Mg(OH)_2_ (filled squares). HIPS and HIPS/Pr show the typical HRR curve of a non-charring material; HIPS/Mg(OH)_2_ and HIPS/Pr/ Mg(OH)_2_, that of residue-forming material. Adding Pr reduces the HRR and THE by flame inhibition; Mg(OH)_2_ reduces the HRR mainly by introducing a barrier.

Taking into account that absorbed water and an alkaline pH value supported the condensed phase action of Pr in PA 66-GF and PBT*-GF, a combination of Pr with Mg(OH)_2_ is proposed in HIPS in order to achieve more than just a superposition. Combining a metal hydroxide with Pr may lead to a reduced amount of flame retardant needed to pass fire tests, or reduce problems such as brittle or reddish-brown materials [118,119]. The flame retardant Mg(OH)_2_ is reported to act as a flame retardant mainly due to dilution and cooling effects caused by the endothermic formation of water in the condensed phase and release of water into the gas phase, as well as by heat barrier effects due to the formation of a surface layer containing MgO [119,120,121,122]. In combination with Pr, Mg(OH)_2_ is expected to act as a source of water release and alkaline. Thus, the combination is expected to induce a condensed phase action of Pr in HIPS. The thermal and thermo-oxidative decomposition of HIPS containing Pr, Mg(OH)_2_ and both flame retardants were studied using TG, TG-FTIR and the residues investigated by XPS and NMR [86,123]. All materials showed one main decomposition step, as radical HIPS scission predominated during anaerobic decomposition. Pr vaporized during the pyrolysis of HIPS/Pr. Mg(OH)_2_ decomposed, released water and formed a barrier of incombustible magnesium oxide in HIPS/Mg(OH)_2_. Using both flame retardants resulted in most of the Pr vaporizing, and in Mg(OH)_2_ combining with some Pr to form stable amorphous magnesium phosphates. Unambiguous proof of glassy Mg-phosphates was obtained by NMR, as mentioned in the literature [123]. The combination of Pr and Mg(OH)_2_ in HIPS led to an increase in inorganic residue but no additional carbonaceous char formation. It became clear that a char-inducing effect of Pr is obtained only in the presence of suitable decomposition pathways of the polymer structure, but is not caused by the mere presence of heteroatoms like oxygen. The most obvious effect of the 15 wt.-% Mg(OH)_2_ used in the cone calorimeter is not the release of incombustible cooling agents such as water, but barrier formation in the condensed phase. HIPS and HIPS/Pr show the typical HRR curve of a non-charring material, whereas HIPS/Mg(OH)_2_ and HIPS/Pr/Mg(OH)_2_ showed the typical HRR curve of a charring—or better, residue-forming—one (Figure 11). 

The clear change in the HRR curve was accompanied by a clear decrease in PHRR, but has no significant influence on the THE. The barrier properties of the inorganic residue layer were clearly improved in comparison to HIPS/Mg(OH)_2_. HRR and PHRR were reduced and the burning time was increased (Figure 11). With respect to the barrier properties, the combination of Pr and Mg(OH)_2_ shows clear synergy, since MgO is replaced by glassy Mg-phosphates. Vaporized Pr significantly reduced the THE/TML through radical trapping in the gas phase and thus also the THE (Figure 11). The combination shows antagonism with respect to flame inhibition. This result corresponds well with the fact that the LOI of the combined HIPS/Mg(OH)_2_/Pr material is characterized by an antagonism compared to HIPS, HIPS/Mg(OH)_2_ and HIPS/Pr (Table 10). However, over all (synergism and antagonism) the fire retardancy of HIPS containing Pr and Mg(OH)_2_ was approximated quite well by a superposition, so that the combination is the far more interesting system with respect to fire retardancy. When low external heat fluxes were used in the cone calorimeter, the observed fire risks approximated the performance of PA 66-GF/Pr. Thus HIPS/Pr/Mg(OH)_2_ is consistently classified as V-1 in the UL 94 (Table 10). This V-1 classification is achieved for a rather low LOI value of 22.8%, which corresponds to the lowest values reported in the literature, like V-0 classification accompanied by a LOI of 22% [79] or V-1 accompanied by 23% [124].

**Table 10:** Flammability of HIPS, HIPS/Pr, HIPS/Mg(OH)_2_ and HIPS/Pr/Mg(OH)_2_.

	HIPS	HIPS/Pr	HIPS/Mg(OH)_2_	HIPS/Pr/Mg(OH)_2_
LOI / % (±1 %)	17.2	22.2	19.5	22.8
UL 94	HB	V-2	HB	V-1

The combination of phosphorus and metal hydrates is appealing, of course, since it tries to use both classes of successful halogen-free flame retardancy approaches. However, even though 15 wt.-% Mg(OH)_2_ showed clear barrier action in the cone calorimeter investigation, neither 15 wt.-% nor Mg(OH)_2_ is the first choice with respect to achieving barrier formation. Further, large amounts of inorganic filler led to brittle materials with the water-releasing or alkaline character of Mg(OH)_2_ decisively destabilizing many polymer structures like PA and polycarbonates [119]. Hence, also small amounts (1 and 5 wt.-%) of nano-dispersed fillers such as AlOOH combined with BDP, or layered silicate with metal hydroxide also have been proposed and used for industrial exploration [125,126,127,128,129,130].

AlPO2 was proposed as a new halogen-free flame retardancy approach for PA 66-GF, but this new phosphorus-containing flame retardant was satisfactorily effective only with rather high loadings of 30 wt.-% [131]. Hence, quite analogous to previous studies, combinations with other flame retardants were proposed [23,132,133,134,135,136,137,138], so that only 10 wt.-% of AlPO2 in combination with melamin polyphosphate (MPP) does the job in polyamides [134,135,139]. Beyond the combination of AlPO2 and MPP, a combination of AlPO2, MPP and zinc borate was suggested and has become the successful commercial product. The propensity of boron compounds to improve flame retardancy efficiency has been reported before [140,141,142]. Zinc borate dehydrates endothermically and vaporizes water, absorbs heat and dilutes fuel [143,144]. Further it can melt to produce glassy layers and change the char properties crucially [142]. The pyrolysis, flammability and fire behavior of PA 66-GF containing AlPO2, MPP, AlPO2 + MPP, and PA 66-GF containing AlPO2 + MPP + zinc borate were investigated [23]. Fire residues were analyzed by means of FTIR, SEM/EDX and NMR. Main decomposition pathways are proposed based on a comprehensive characterization of thermal decomposition (TG, kinetics), evolved pyrolysis products (TG-FTIR) and the residue (FTIR-ATR). A clear interaction between the various additives and the polymer structure was observed during decomposition. As a result the polymer decomposition was shifted to lower temperatures. The effect is greatest when adding MPP followed by AlPO2 + MPP + zinc borate and AlPO2. The effect is smallest when adding MPP + AlPO2. The Lewis acid/base interactions between the flame retardants, the amide unit, and the metal ions control the decomposition. Adding MPP, results in the strongest Lewis acid/base interaction between MPP and PA 66. If MPP and AlPO2 or MPP, AlPO2 and zinc borate are combined, a strong reaction occurs between the flame retardants. The addition of MPP to PA 66-GF shows a fuel-dilution effect in the gas phase and a phosphate barrier in the condensed phase. Adding AlPO2 resulted in a predominant vaporization and release of phosphinic acid, resulting in very effective flame inhibition. Correspondingly, PA 66-GF/AlPO2 shows a very high LOI of 37.9%. However, despite this high LOI, this composite does not achieve a V-0 classification, but exhibits only HB behavior. Combining both additives alters these flame-retardancy mechanisms. The flame retardants react with each other to form aluminium phosphate. Some charring and an effective barrier layer of aluminium phosphate were observed and dominated the fire protection; the flame inhibition due to vaporized phosphinic acid and the fuel dilution due to vaporized melamine decomposition products become less important. The combination of AlPO2 and MPP and zinc borate further improves the barrier in the condensed phase. A zinc, boron, aluminium phosphate layer is formed, which was clearly identified by ^11^B, ^31^P, and ^27^Al solid-state NMR [22]. These results correspond to the studies on the effect of zinc borate on the thermal degradation of APP, in which the formation of boron polyphosphate and zinc polyphosphate was also observed [145]. The altered decomposition controls the fire retardancy mechanisms. The predominant flame-inhibition effect by the phosphinates in PA 66-GF/AlPO2 is replaced by a strong barrier effect of aluminium and boron phosphates in PA 66-GF/AlPO2/MPP and PA 66-GF/AlPO2/MPP/zinc borate, respectively. The flame inhibition effect is reduced. The aluminium and boron phosphates influence the stability of the residue. Inhomogeneous and complex structured residues are formed under fire [22], characterized by functional and effective barrier layers. The aluminium and boron phosphates form a glassy, rather closed surface layer. This surface layer, together with the hollow area beneath, which is stabilized by the glass fibers, protects underlying material through insulation. In fact self-extinction of PA 66-GF/AlPO2/MPP/zinc borate occurs in cone calorimeter test with a low irradiation. Correspondingly, PA 66-GF/AlPO2/MPP/zinc borate achieves a V-0 classification. The LOI is 33.3%. 

The UL 94 and the LOI results are not correlated when PA 66-GF, PA 66-GF/MPP, PA 66-GF/AlPO2, PA 66-GF/MPP/AlPO2 and PA 66-GF/AlPO2/MPP/zinc borate are compared with each other, because the mechanisms change so crucially. The residues are especially dissimilar. In the stabilization of the inhomogeneously structured char, the presence of glass fibers was concluded to be crucial, in accordance with the results for other glass fiber reinforced thermoplastic systems [111,146]. In fact, the PA 66-GF/ AlPO2/MPP/zinc borate system is characterized by a kind of residue design. 

## 3. Conclusions

Systematic studies working out the structure-property relationships, like the ones presented here in the examples, are, as yet, still rare. Indeed, a deeper understanding of the actual mechanisms often remains in its infancy, as do the fundamentals for directed development. This review is actually characterized by a different approach and represents complementary points of view for future discussion. It is based on a distinctively systematic comparison of materials and focuses on the mechanisms controlling pyrolysis and flame retardancy in both the condensed and the gas phases.

Thus, the paper aims to make a valuable contribution towards a better understanding of phosphorus-containing flame retardants and thereby provide a foundation for the directed design of new flame retarded polymeric materials. A detailed understanding of the decomposition behavior and the resulting flame retardancy mechanisms, opens the door to a tailored development thanks to the targeted modification of the decomposition pathways and hence the appearance and efficiency of flame retardancy mechanisms. Together with a comprehensive assessment of fire behavior and flame retardancy as regards different fire properties, and the response in different fire scenarios, it yields useful guidelines for the development of future systems.
